# Osimertinib in NSCLC With Atypical *EGFR*-Activating Mutations: A Retrospective Multicenter Study

**DOI:** 10.1016/j.jtocrr.2022.100459

**Published:** 2023-01-10

**Authors:** Jingran Ji, Jacqueline V. Aredo, Andrew Piper-Vallillo, Laura Huppert, Julia K. Rotow, Hatim Husain, Susan Stewart, Rosemary Cobb, Heather A. Wakelee, Collin M. Blakely, Melisa L. Wong, Matthew A. Gubens, Mohammad H. Madani, Subba R. Digumarthy, Caroline McCoach, Zofia Piotrowska, Joel W. Neal, Jonathan W. Riess

**Affiliations:** a City of Hope Comprehensive Cancer Center, Duarte, California; b UC Davis Comprehensive Cancer Center, Sacramento, California; c Stanford Cancer Institute, Stanford, California; d Massachusetts General Hospital Cancer Center, Boston, Massachusetts; e UCSF Helen Diller Comprehensive Cancer Center, San Francisco, California; f Dana-Farber Cancer Institute, Boston, Massachusetts; g UCSD Moores Comprehensive Cancer Center, La Jolla, San Diego, California

**Keywords:** Osimertinib, Non–small cell lung cancer, Atypical *EGFR* mutation, L861Q, G719X

## Abstract

**Introduction:**

*EGFR* mutations drive a subset of NSCLC. Patients harboring the common *EGFR* mutations, deletion of exon 19 and L858R, respond well to osimertinib, a third-generation tyrosine kinase inhibitor. Nevertheless, the effect of osimertinib on NSCLC with atypical *EGFR* mutations is not well described. This multicenter retrospective study evaluates the efficacy of osimertinib among patients with NSCLC harboring atypical *EGFR* mutations.

**Methods:**

Patients with metastatic NSCLC treated with osimertinib, harboring at least one atypical *EGFR* mutation, excluding concurrent deletion of exon 19, L858R, or T790M mutations, from six U.S. academic cancer centers were included. Baseline clinical characteristics were collected. The primary end point was the time to treatment discontinuation (TTD) of osimertinib. Objective response rate by the Response Evaluation Criteria in Solid Tumors version 1.1 was also assessed.

**Results:**

A total of 50 patients with NSCLC with uncommon *EGFR* mutations were identified. The most frequent *EGFR* mutations were L861Q (40%, n = 18), G719X (28%, n = 14), and exon 20 insertion (14%, n = 7). The median TTD of osimertinib was 9.7 months (95% confidence interval [CI]: 6.5–12.9 mo) overall and 10.7 months (95% CI: 3.2–18.1 mo) in the first-line setting (n = 20). The objective response rate was 31.7% (95% CI: 18.1%–48.1%) overall and 41.2% (95% CI: 18.4%–67.1%) in the first-line setting. The median TTD varied among patients with L861Q (17.2 mo), G719X (7.8 mo), and exon 20 insertion (1.5 mo) mutations.

**Conclusions:**

Osimertinib has activity in patients with NSCLC harboring atypical *EGFR* mutations. Osimertinib activity differs by the type of atypical *EGFR*-activating mutation.

## Introduction

*EGFR* mutations are present in 10% to 15% of NSCLC, occurring in higher frequency in lung adenocarcinoma, patients with light or never smoking history, women, and those of East Asian descent.^1^, ^2^, ^3^ *EGFR* mutations are heterogeneous and variable in frequency, with the in-frame deletion of exon 19 (Ex19del) and the L858R point mutation comprising approximately 80% to 85% of all *EGFR* mutations.^1^^,^^4^ The remaining 10% to 15% consist of a heterogeneous group of mutations in *EGFR* including exon 20 insertions (approximately 5%–12%), G719X (4%), L861Q (2%), and S768I (1%), among others.^4^^,^^5^

In advanced *EGFR*-mutant NSCLC harboring Ex19del or L858R, EGFR tyrosine kinase inhibitors (TKIs) are the standard first-line treatment. First- and second-generation TKIs such as gefitinib, erlotinib, afatinib, and dacomitinib were found to be highly active in these *EGFR*-activating mutations compared with standard chemotherapy.^6^, ^7^, ^8^, ^9^, ^10^, ^11^ Osimertinib is a third-generation TKI that irreversibly binds the EGFR receptor, including *EGFR* T790M (the most common resistance mechanism to first- and second-generation EGFR TKIs).^12^^,^^13^ In the first-line setting, the FLAURA trial compared osimertinib with erlotinib or gefitinib in advanced *EGFR*-mutant NSCLC with canonical Ex19del and L858R mutations. The results revealed a substantial improvement in median progression-free survival (mPFS) and overall survival (OS) that led to the approval of osimertinib as the preferred first-line therapy for NSCLC harboring *EGFR* Ex19del or L858R mutations.^14^^,^^15^

Afatinib, a second-generation EGFR TKI, is approved by the Food and Drug Administration (FDA) for the uncommon *EGFR* mutations L861Q, G719X, and S768I (based on pooled analysis of LUX-Lung 2, LUX-Lung 3, and LUX-Lung 6 clinical trials) with an estimated mPFS of 10.7 months and an objective response rate (ORR) of 71.1%.^16^ More recently, a retrospective multicenter analysis in Germany evaluated EGFR TKIs for uncommon mutations and afatinib was the most frequently used TKI with a mPFS of 12.0 months for L861Q, G719X, and S768I mutations.^17^ A retrospective study of erlotinib in this setting also revealed a median time to progression of 3 months and a response rate of 27%.^18^ One study in Taiwan evaluated specifically G719X, L861Q, and S768I mutations and found a mPFS of 7.7 months and an ORR of 40.5%.^6^ For patients with lung cancer harboring L861Q and G719X mutations, gefitinib was found to have a median OS of 12 months, compared with a median OS of 28.4 months when using gefitinib for patients harboring the common mutations (*p* = 0.002). The mPFS was 2.2 months versus 11.4 months (*p* < 0.001), and ORR was 20% versus 76%, respectively (*p* = 0.017).^19^ In another study that included all mutations other than L858R, T790M, Ex19del, and exon 20 insertion, those who harbored G719X or L861X mutations had a response rate of 57.1% and a mPFS of 6.0 months. Other uncommon mutations had a response rate of 20.0% and a mPFS of 1.6 months.^20^

There are less data on osimertinib in patients harboring atypical mutations, representing approximately 15% of *EGFR*-activating mutations. Cho et al. in South Korea conducted the first prospective phase 2 trial with osimertinib as first-line therapy in 37 patients harboring uncommon *EGFR* mutations.^21^ They studied patients with uncommon *EGFR*-activating mutations excluding exon 19, L858R, T790M, and exon 20 insertion with a primary end point of objective response rate. In their cohort of 37 patients, the ORR was 50% (95% CI: 33–67) with a mPFS of 8.2 months (95% CI: 5.9–10.5). Notably, they found that within the small cohort of patients with L861Q alterations (n = 9), PFS was modestly improved (mPFS = 15.2 mo).^21^ Given the small sample size and heterogeneity of uncommon *EGFR* mutations, additional data are needed to further elucidate the clinical efficacy of osimertinib in this patient population including in a U.S. population.

Furthermore, among uncommon EGFR mutations, *EGFR* exon 20 insertions represent a sizable (5%–12% of cases) albeit heterogeneous subset with more than 60 unique insertions described.^4^ The bifunctional EGFR/MET monoclonal antibody amivantamab and the EGFR TKI mobocertinib are currently approved as second-line therapy.^22^^,^^23^ Nevertheless, osimertinib has preclinical and limited clinical data of activity in *EGFR* exon 20 insertion.^24^ In a small phase 2 trial by van Veggel et al.^25^ studying osimertinib in any line of therapy in EGFR exon 20 insertion mutations, patients were treated with osimertinib 80 or 160 mg daily dosing. The ORR was 5%, and mPFS was 3.6 months.^25^ A more recent phase 2 trial at the 160 mg dose of osimertinib revealed an ORR of 25% and a mPFS of 9.7 months.^26^^,^^27^

Less is known about the efficacy of osimertinib for other atypical *EGFR* mutations. This study describes the pooled experience from six academic medical centers evaluating efficacy of osimertinib among a real-world population of patients with lung cancers harboring atypical *EGFR*-activating mutations.

## Materials and Methods

### Design and Patient Selection

This was a multicenter, single-arm, retrospective study approved on institutional review board protocols at six U.S. National Cancer Institute–designated comprehensive cancer centers (University of California, Davis, Stanford, Massachusetts General Hospital, Dana Farber, University of California, San Francisco, CA and University of California, San Diego, CA). Eligible patients had metastatic NSCLC treated with osimertinib for at least one atypical *EGFR* mutation, excluding those with concurrent L858R, Ex19del, or T790M mutations. Included patients were adults (≥18 y of age), with next-generation sequencing–confirmed atypical EGFR-mutant NSCLC and biopsy-confirmed NSCLC. Previous or subsequent chemotherapy, first- or second-generation EGFR TKI therapy, and radiation therapy were also permitted.

### Objectives

Time to treatment discontinuation (TTD) on osimertinib as a measure of clinical benefit and as a surrogate for PFS was the primary end point in this retrospective analysis.^28^ This was defined as the time interval during which a patient was started on osimertinib to discontinuation of therapy for any reason. In eligible patients with measurable disease, a secondary end point was ORR, which was defined as the percentage of patients who achieved a complete response or a partial response as defined by the Response Evaluation Criteria in Solid Tumors version 1.1.

### Statistical Analysis

TTD was analyzed using the Kaplan-Meier method through the Statistical Package for the Social Sciences version 25 (IBM Corp., Armonk, NY). Data are expressed using median values and associated 95% confidence intervals (CIs). Log rank tests were used for comparisons between survival curves. The 95% CIs for ORRs were calculated with the Copper-Pearson exact method.

## Results

### Demographics

A total of 50 patients were identified among six academic cancer centers in this retrospective analysis. Patient characteristics are listed in Table 1. The median age was 65 (range: 44–83) years, and 37 (74.0%) were of female sex. There were 42 patients who had Eastern Cooperative Oncology Group score of 0 to 1 (84.0%), 21 (42.0%) were never smokers, and 50 (100%) had adenocarcinoma. In addition, 36 (72.0%) had stage IV disease at presentation. There were 20 patients (40.0%) who received first-line osimertinib. The most common atypical *EGFR* mutation was L861X (40.0%) followed by G719X (28.0%) (Table 2). Two patients had S768I (4.0%) and seven patients had exon 20 insertion (14.0%). Ten patients had other *EGFR* mutations (20.0%).

**Table 1:** Baseline Participant Characteristics

Characteristic	
Median age at diagnosis (range), y	65 (range 44–83)
Sex (%)	
Men	13 (26.0)
Women	37 (74.0)
ECOG (%)	
0	14 (28.0)
1	28 (56.0)
2	3 (6.0)
3	1 (2.0)
Unknown	4 (8.0)
Smoking status (%)	
Never smoked	21 (42.0)
Former smoker	29 (58.0)
Current smoker	0 (0)
Average pack-year (if smoker)	14.1
Histology (%)	
Adenocarcinoma	50 (100)
Squamous cell carcinoma	0 (0)
Line of therapy (%)	
First line	20 (40.0)
Second line	10 (20.0)
Above or equal to third line	20 (40.0)
Prior EGFR TKI	27 (54.0)
Race (%)	
White	29 (58.0)
Asian	13 (26.0)
Hispanic	1 (2.0)
Black	2 (4.0)
Other	5 (10.0)
Stage at diagnosis (%)	
I	6 (12.0)
II	2 (4.0)
III	6 (12.0)
IV	36 (72.0)

ECOG, Eastern Cooperative Oncology Group; TKI, tyrosine kinase inhibitor.

**Table 2:** Mutation Distribution of Participants

EGFR Mutation(s)	N (%)
L861X (%)	20 (40.0)
L861Q	14 (28.0)
L816Q + L833F	2 (4.0)
L861Q + K852N	1 (2.0)
L861Q + G719A	1 (2.0)
L861Q + L858M	1 (2.0)
L861R + V774M	1 (2.0)
G719X (%)	14 (28.0)
G719A	7 (14.0)
G719D	1 (2.0)
G719S	1 (2.0)
G719A + K757M	1 (2.0)
G719A + E709A	1 (2.0)
G719S + E709A	1 (2.0)
G719A + L861Q	1 (2.0)
G719A + S768I	1 (2.0)
S768I (%)	2 (4.0)
S768I	1 (2.0)
S768I + G719A	1 (2.0)
Other mutations (%)	
Exon 20 insertion	7 (14.0)
V774M	2 (4.0)
L747P	1 (2.0)
Exon 18–25 duplication	1 (2.0)
Exon 18 deletion	1 (2.0)
Exon 19 insertion	1 (2.0)
G711A	1 (2.0)
H773R	1 (2.0)
L833V + H835L	2 (4.0)

### Efficacy

**Figure 1:**
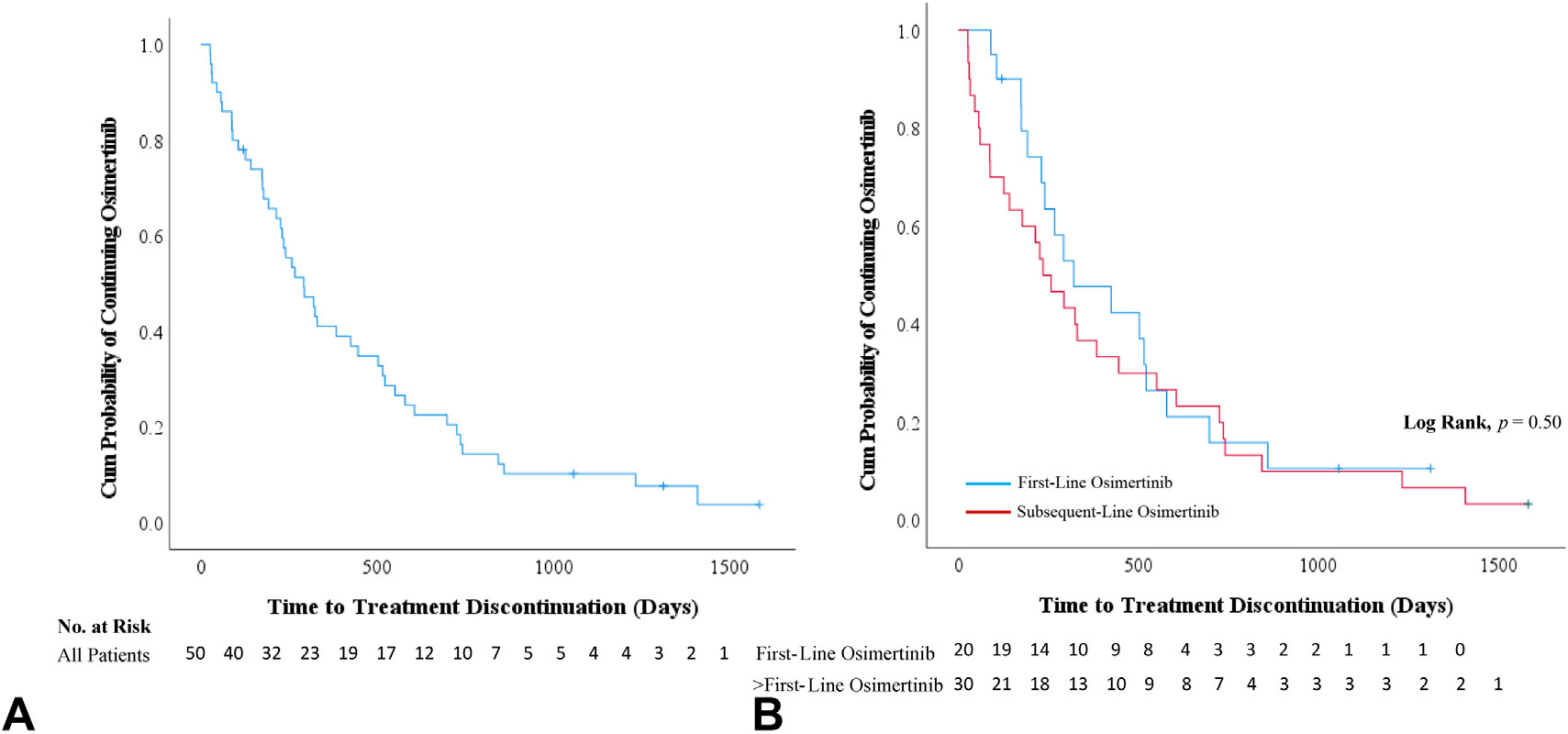
Kaplan-Meier analysis of time to discontinuation of osimertinib in (*A*) overall population and in (*B*) first- and subsequent-line settings.

**Figure 2:**
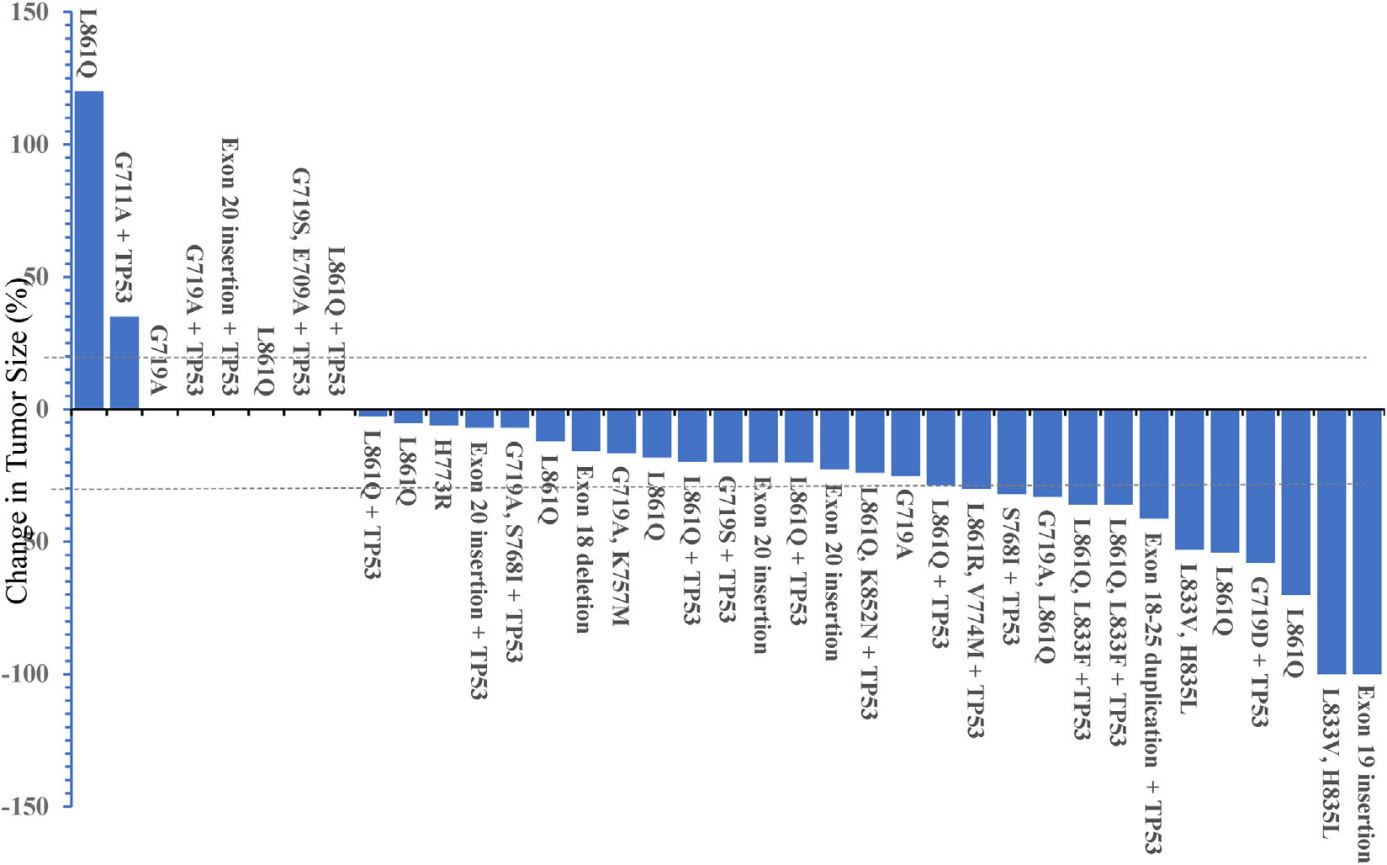
Best percentage change in target lesions among patients with atypical *EGFR* mutations. The upper dashed line marks the threshold for progressive disease at 20% increase in the sum of the longest diameter of target lesions. The lower dashed line at −30% marks the threshold for partial response.

Overall, patients harboring atypical *EGFR* mutations had a median TTD of osimertinib of 9.7 months (95% CI: 6.5–12.9 mo) (Fig. 1*A* and Table 3). In patients with uncommon *EGFR* mutations who received osimertinib as first-line therapy, the median TTD was 10.7 months (95% CI: 3.2–18.1 mo). Patients who received osimertinib as a subsequent line of therapy had a median TTD of 7.8 months (95% CI: 4.3–11.4 mo) (Fig. 1*B*). The ORR in the overall cohort was 31.7% (95% CI: 18.1%–48.1%) (Fig. 2). The ORR in patients who received osimertinib as first-line therapy was 41.2% (95% CI: 18.4%–67.1%). Excluding patients harboring an exon 20 insertion (n = 7), the median TTD of osimertinib was 10.8 months (95% CI: 4.15–17.5 mo) overall and 14.2 months (95% CI: 3.7–24.7 mo) in the first-line setting. The ORR was 36.1% (95% CI: 20.8%–53.8%) and 46.7% (95% CI: 21.3%–73.4%), respectively.

**Table 3:** Median Time on Osimertinib and Objective Response Rates by Uncommon Mutation

Mutations	Median Time on Osimertinib Mo (95% CI), Total No.		Objective Response % (95% CI), Total No.	
	Overall	First Line Only	Overall	First Line Only
Total	9.7 (6.5–12.9), n = 50	10.7 (3.2–18.1), n = 20	31.7% (18.1–48.1), n = 41	41.2% (18.4–67.1), n = 17
S768I+G719X+L861Q	10.8 (2.7–19.0), n = 33	17.2 (5.5–27.1), n = 15	32.1% (15.9–52.4), n = 28	38.4% (13.9–68.4), n = 14
G719X	7.8 (0.6–15.0), n = 14	5.8 (1.1–15.0), n = 4	10.0% (3.0–44.5), n = 10	33.3% (8.0–90.6), n = 3
L861Q	17.2 (3.3–31.1), n = 18	19.3 (13.2–25.4), n = 10	41.2% (18.4–67.1), n = 17	40.0% (12.2–73.8), n = 10
Exon 20 insertion	1.5 (0.4–2.6), n = 7	8.0 (CI not calculable), n = 2	0.0% (0.0–52.2), n = 5	0.0% (0.0–84.2), n = 2
Other mutations	7.7 (0–15.9), n = 9	7.7 (0–18.1), n = 3	30.0% (9.9–81.6), n = 7	100.0% (2.5–100.0), n = 1
TP53 mutant	5.9 (2.4–9.5), n = 23	8.9 (2.2–16.5), n = 8	27.7% (9.7–53.5), n = 18	33.3% (4.3–77.7), n = 6
TP53 wild-type	14.8 (5.1–24.6), n = 27	14.1 (2.2–26.1), n = 12	34.7% (16.4–57.3), n = 23	45.5% (16.7–76.6), n = 11
Asian	7.8 (4.6–11.1), n = 13	6.4 (2.7–10.1), n = 6	11.1% (0.3–48.2), n = 9	0.0% (0.0–52.2), n = 5
Non-Asian	12.8 (6.4–19.2), n = 37	16.8 (7.6–24.6), n = 14	37.5% (21.1–56.3), n = 32	58.3% (27.7–84.8), n = 12

CI, confidence interval.

**Figure 3:**
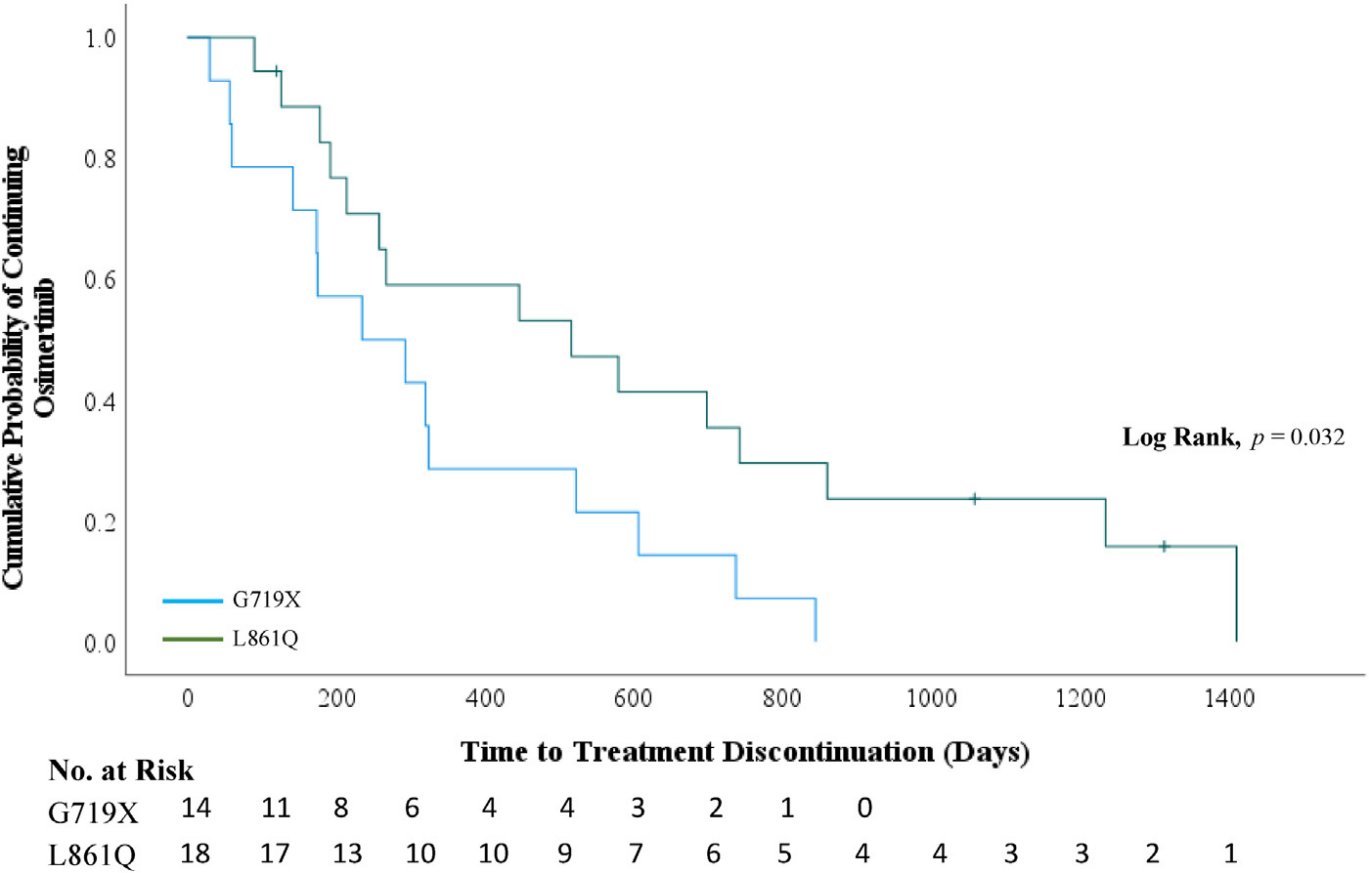
Kaplan-Meier analysis of time on osimertinib in the overall population when comparing patients harboring L861Q mutations (green line) versus those harboring G719X mutations (blue line).

When looking at specific mutations (Table 3), comparing osimertinib in both the first and subsequent-line setting for the two most common atypical mutations G719X and L861Q, patients with the G719X mutation (n = 14) had a significantly shorter median TTD of 7.8 months (95% CI: 0.6–15.0 mo) compared with patients with the L861Q mutation (n = 18) with a median TTD of 17.2 months (95% CI: 3.2–31.1 mo, log rank, *p* = 0.032) (Fig. 3). In the first-line setting, patients with the G719X mutation had a median TTD of 5.8 months (n = 4). Patients with the L861Q mutation had a median TTD of 19.3 months (n = 10). Patients with exon 20 insertion (n = 7) had a median TTD of 1.5 months overall (95% CI: 0.4–2.5 mo) and 8.0 months in the first-line setting (n = 2). Patients with other exceedingly rare mutations also responded to osimertinib with variable outcomes (Supplementary Table 1). Of note, one patient with S768I had a partial response and was on osimertinib for 24.2 months. One patient whose tumor harbored an EGFR exon 19 insertion had a partial response and was on osimertinib for 16.8 months. Another patient with L883V and H835L comutations also had a partial response and continues to be on osimertinib for more than 52.8 months.

Patients who had concurrent *TP53* mutations (n = 23) had shorter median TTD of 5.9 months (95% CI: 2.4–9.5 mo) compared with those with wild-type *TP53* (n = 27) who had a median TTD of 14.8 months (95% CI: 5.1–24.6 mo, log rank, *p* = 0.012) (Table 3 and Supplementary Fig. 1). Furthermore, non-Asian patients with atypical *EGFR* mutations (n = 37) had a median TTD of 12.8 months (95% CI: 6.4–19.2 mo) and the Asian patients (n = 13) had a median TTD of 7.8 months (95% CI: 4.6–11.1 mo), though this difference was not statistically significant (log rank, *p* = 0.123) (Table 3 and Supplementary Fig. 2).

## Discussion

This is the first and largest known retrospective study in the United States investigating the clinical activity of the third-generation EGFR TKI osimertinib in uncommon *EGFR* mutations where osimertinib is not FDA approved but may have efficacy. In this multicenter retrospective analysis, the median TTD for osimertinib was 9.7 months in the overall cohort and 10.7 months in the first-line setting. Patients with tumors harboring L861Q had longer TTD for osimertinib compared with other uncommon mutations, especially in the first-line setting. One patient with *EGFR* exon 19 insertion (1% of all *EGFR* mutations that function similarly to *EGFR* Ex19del mutations) was on therapy for 16.8 months.^29^ Among non-Asian patients, TTD of first-line osimertinib was longer compared with Asian patients, though the number of patients was limited and the difference was not statistically significant (*p* = 0.123). Interestingly, in a post hoc subset analysis of the FLAURA study that randomized patients with *EGFR* Ex19del and *EGFR* L858R to osimertinib versus gefitinib or erlotinib, OS was improved in non-Asian patients, which was not noted in the subset of treated Asian patients.^14^ Similarly to common EGFR mutations, the presence of a p53 comutation was associated with inferior clinical outcomes to osimertinib.^30^

TTD on osimertinib was used as the primary end point of efficacy as a practical surrogate of PFS in this retrospective multicenter analysis. TTD has been evaluated as a clinical end point among metastatic NSCLC trials submitted to the FDA, which included four EGFR TKI trials in *EGFR*-mutant NSCLC, and highly correlated with PFS.^28^ TTD can be an overestimate compared with PFS (approximately 2 mo more in pooled *EGFR*-mutant lung cancer studies), but it also may reflect a more real-world approach where TKI is often continued in the presence of clinical benefit despite potential Response Evaluation Criteria in Solid Tumors progression.^31^

Although it is difficult to compare results across studies, our estimate in the real-world setting of median TTD on first-line osimertinib reflects approximately half the time compared with the PFS of osimertinib in patients harboring the Ex19del and L858R, though substantial variation is observed depending on the particular uncommon activating mutations.^32^ For instance, L861Q seemed to respond similarly to these canonical EGFR mutations. Though TTD may be an overestimate as a surrogate of PFS, our median TTD of 10.7 months is in range to that of the prospective trial by Cho et al. which reported a mPFS of 8.1 months when looking at osimertinib as first-line therapy. We also found that heterogeneous activity depends on the specific mutational subtype with patients harboring L861Q having the longest time on therapy, especially in the first line, again comparable with results found in Cho et al.^21^ Furthermore, we add to the existing data by having a large proportion of non-Asian patients and estimate a median TTD of first-line osimertinib of 12.8 months in this patient population.

Our results also reveal that patients with NSCLC harboring atypical mutations may respond differently to osimertinib compared with afatinib. Afatinib is FDA approved for the treatment of metastatic NSCLC harboring G719X, L861Q, and S768I activating mutations on the basis of pooled analysis of the LUX-Lung 2, 3, and 6 data with mPFS of 13.8 months, 8.2 months, and 14.7 months, respectively.^16^ In a recent pooled analysis of 693 patients with uncommon *EGFR* mutations treated with afatinib using real-world data, median time to treatment failure was 10.8 months. Patients harboring G719X, L861Q, and S768I had median time to treatment failure of 14.7 months, 10.0 months, and 15.6 months, respectively.^33^ In our first-line analysis of osimertinib, median TTD was 5.8 months and 19.3 months for G719X and L861Q, respectively. We did not have sufficient patients to calculate a TTD estimate for patients harboring S768I, but one patient did have 24.2 months on osimertinib.

Preclinical in vivo and in vitro studies have found antitumor activity of osimertinib in the uncommon mutations G719X, L861Q, and S768I.^24^ Nevertheless, in a structure–function-based classification of *EGFR*-mutant NSCLC, those with P-loop and alpha-C-helix compressing mutations in *EGFR* such as G719X were predicted to have inferior outcomes to third-generation EGFR TKI *in silico* and *in vivo*. Indeed, we observed TTD in G719X which was inferior compared with that in other structural subgroups of *EGFR* mutations which included such as L861Q and exon 19 insertions.^34^ Our data among six U.S. cancer centers suggest that osimertinib as first-line treatment of metastatic NSCLC harboring L861Q is comparable with the more common L858R and Ex19del mutations and that the activity of osimertinib seems less robust in G719X. Our U.S. data further support the results of the study of Cho et al., which revealed mPFS of 15.2 months and 8.2 months for L861Q and G719X, respectively.

The ORR to osimertinib in uncommon *EGFR* mutations was 31.7% overall, 41.1% in the first line and 25% in the subsequent lines. Excluding exon 20 mutations which respond suboptimally to the 80 mg dose of osimertinib, the ORR became 36.1% overall and 46.7% in the first-line setting. Our response rate in the first-line setting, after excluding exon 20 insertions, is comparable with Cho et al. with an ORR of 50%, which also excluded patients with exon 20 insertions. We suspect that the drop in response in subsequent lines of therapy is due to both patients who received treatment other than EGFR TKIs before osimertinib in the overall subgroup (54.0%) and the fact that several patients were either lost to follow-up, admitted to hospice, or taken off treatment without subsequent imaging. Another limitation of our study is the small sample size, with only 20 patients receiving first-line osimertinib, which limits the power to calculate statistical significance when comparing between subgroups. The heterogeneity of data collection between institutions and variation in assessment of response may have also been a limitation. The retrospective nature of the study and the use of TTD further limit the ability to draw direct comparisons.

Patients harboring exon 20 insertion mutations did considerably worse than the rest of the cohort with a TTD of 1.5 months, though the two patients with *EGFR* exon 20 insertion NSCLC who received osimertinib in the first-line setting had an average TTD of 8 months. *EGFR* exon 20 insertions represent a sizable (5%–12% of cases) albeit heterogeneous subset with more than 60 unique insertions described, and for which several novel therapies are being investigated.^4^ Historically, patients with exon 20 insertions had an ORR of 9%, a mPFS of 2.7 (95% CI: 1.8–4.2) months, and a median OS of 9.2 (95% CI: 4.1–14.2) months when treated with afatinib in the second-line setting.^16^ Activity with osimertinib at the 80 mg dose seems to be limited as well. Previously, 21 patients with pretreated NSCLC with an exon 20 insertion were prospectively treated with osimertinib and found to have an ORR of 5% and a mPFS of 3.6 (95% CI: 2.6–4.5) months.^25^ More recently, a dose of osimertinib at 160 mg was used in 21 patients with pretreated NSCLC harboring an exon 20 insertion which led to a higher ORR of 25% and a median duration of response of 5.7 (95% CI: 4.73–not applicable) months.^26^ Another phase 2 trial at the 160 mg dose of osimertinib had an ORR of 28% and a mPFS of 6.8 months.^27^ Although there may be some activity in *EGFR* exon 20 insertion NSCLC, particularly at the 160 mg dose, it is not comparable with the efficacy of osimertinib in patients with the canonical Ex19del and L858R activating mutations in *EGFR*. The bispecific EGFR-MET monoclonal antibody amivantamab and the EGFR TKI mobocertinib are currently approved as second-line therapy.^22^^,^^23^

Finally, our study included a few patients with exceedingly rare mutations who had variable responses to osimertinib. One patient with an exon 19 insertion had a TTD of 16.8 months, consistent with previous reports suggesting that these patients have similar benefit to EGFR TKIs as patients harboring the more common deletions in *EGFR* exon 19.^35^ Another patient with an L833V plus H835L compound heterozygote *EGFR* mutation had a TTD of 52.8+ months (ongoing), which has also been reported to have favorable response to osimertinib.^36^ Some had more resistant mutations such as V774M (TTD of 3.5 mo) and L747P (7.7 mo), comparable with previously described case reports.^37^^,^^38^ Others had lesser known mutations such as G711A (TTD 12.8 mo) and H733R (TTD 18.3 mo). This heterogeneous, mutation-specific response supports the work of Robichaux et al.^34^ who identified four distinct structural subgroups of EGFR which correlated with response to treatment, independent of the involved exon.^29^ Their work suggests that a structure and function–based approach to developing future treatment for *EGFR*-mutant NSCLC may be more advantageous than the traditional exon-based strategy.

In summary, to our knowledge, this is the one of the largest retrospective reviews of osimertinib used to treat patients with atypical *EGFR*-mutant NSCLC. Our data suggest that osimertinib has activity in atypical *EGFR* mutations, although the first-line clinical benefit seems lower in this multicenter U.S. cohort than that with patients harboring Ex19del or L858R mutations. We also propose that even among patients with atypical *EGFR* mutations, the mutations are heterogeneous with some uncommon mutations responding better to therapy than others, particularly *EGFR* L861Q, *EGFR* exon 19 insertion, *EGFR* S768I, *EGFR* L833V plus H835L, and *EGFR* H733R. Our results prompt further investigation of osimertinib in this patient population, particularly additional prospective trials in the United States and international patients with *EGFR*-activating mutations.

## CRediT Authorship Contribution Statement

**Jingran Ji**: Conceptualization, Data curation, Formal analysis, Investigation, Methodology, Project administration, Visualization, Writing—original draft, Writing—review and editing.

**Jacqueline V. Aredo**: Investigation, Data curation, Writing—review and editing.

**Andrew Piper-Vallillo**: Investigation, Data curation, Writing—review and editing.

**Laura Huppert**: Investigation, Data curation, Writing—review and editing.

**Julia K. Rotow**: Investigation, Data curation, Writing—review and editing.

**Hatim Husain**: Investigation, Data curation, Writing—review and editing.

**Susan Stewart**: Conceptualization, Data curation, Methodology, Writing—review and editing.

**Rosemary Cobb**: Investigation, Data curation, Writing—review and editing.

**Heather A. Wakelee**: Investigation, Data curation, Writing—review and editing.

**Collin M. Blakely**: Investigation, Data curation, Writing—review and editing.

**Melisa L. Wong**: Investigation, Data curation, Writing—review and editing.

**Matthew A. Gubens**: Investigation, Data curation, Writing—review and editing.

**Mohammed H. Madani**: Investigation, Writing—review and editing.

**Subba R. Digumarthy**: Investigation, Writing—review and editing.

**Caroline McCoach**: Investigation, Data curation, Writing—review and editing.

**Zofia Piotrowska**: Investigation, Data curation, Writing—review and editing.

**Joel W. Neal**: Investigation, Data curation, Writing—review and editing.

**Jonathan W. Riess**: Supervision, Conceptualization, Data curation, Formal analysis, Investigation, Methodology, Project administration, Visualization, Writing—original draft, Writing—review and editing.
