# Supplementary material for: Osimertinib in NSCLC With Atypical EGFR-Activating Mutations: A Retrospective Multicenter Study
Source: JTO Clin Res Rep. 2023 Jan 10;4(3):100459. doi: 10.1016/j.jtocrr.2022.100459 (PMC9984841; doi:10.1016/j.jtocrr.2022.100459)

**SUPPLEMENTAL MATERIALS**

**Osimertinib in Non-Small Cell Lung Cancer (NSCLC) with Atypical *EGFR* Activating Mutations: A Retrospective Multicenter Study**

Jingran Ji, Jacqueline V. Aredo, Andrew Piper-Vallillo, Laura Huppert, Julia K. Rotow, Hatim Husain, Susan Stewart, Rosemary Cobb, Heather A. Wakelee, Collin Blakely, Melisa L. Wong, Matthew A. Gubens, Mohammed H. Madani, Subba R. Digumarthy, Caroline McCoach, Zofia Piotrowska, Joel W. Neal, Jonathan W. Riess

**Contents**:

**Supplemental Table 1**. Time on Osimertinib among Patients with Rare Atypical Mutations.

**Supplemental Figure 1**. Kaplan-Meier analysis of time on osimertinib in the overall population when comparing patients harboring *TP53* mutations (green line) versus those who were *TP53* wild type (blue line).

**Supplemental Figure 2**. Kaplan-Meier analysis of time on osimertinib in the overall population when comparing non-Asian (green line) versus Asian (blue line) patients.

**Supplemental Table 1**. Time on Osimertinib among Patients with Rare Atypical Mutations.

|  | **Line of Therapy for Osimertinib** | **Time on Osimertinib (months)** |
| --- | --- | --- |
| **S768I** | 4 | 24.2 |
| **S768I + G719A** | 4 | 4.7 |
| **Exon 18 Deletion** | 3 | 2.9 |
| **Exon 18-25 Duplication** | 5 | 2.9 |
| **Exon 19 Insertion** | 1 | 16.8 |
| **G711A** | 4 | 12.8 |
| **H773R** | 2 | 18.3 |
| **L747P** | 1 | 7.7 |
| **L833V + H835L** | 1 | 14.1 |
| **L833V + H835L** | 3 | 52.8 (ongoing) |
| **V774M** | 1 | 3.5 |

**Supplemental Figure 1**. Kaplan-Meier analysis of time on osimertinib in the overall population when comparing patients harboring *TP53* mutations (green line) versus those who were *TP53* wild type (blue line).


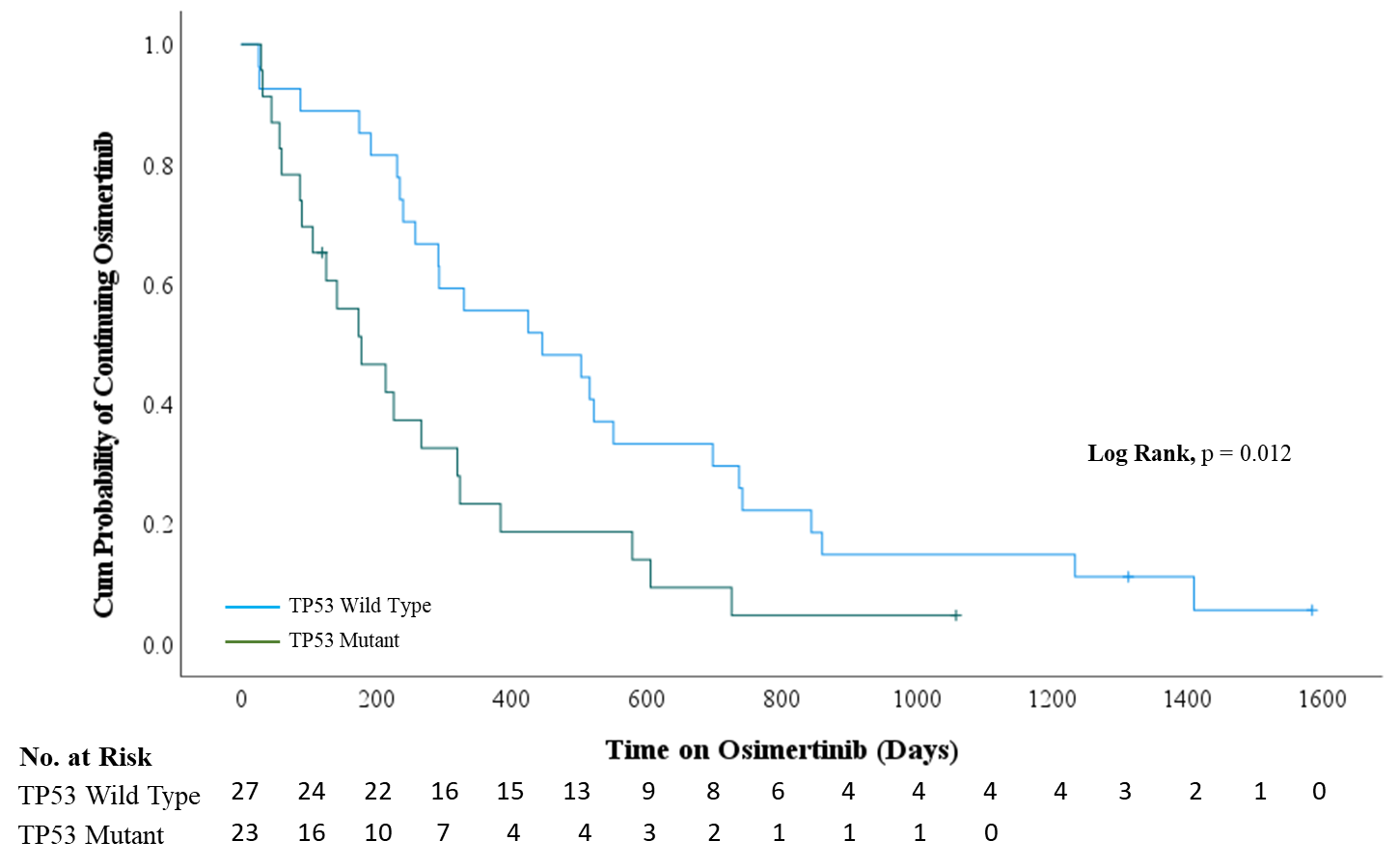


**Supplemental Figure 2**. Kaplan-Meier analysis of time on osimertinib in the overall population when comparing non-Asian (green line) versus Asian (blue line) patients.


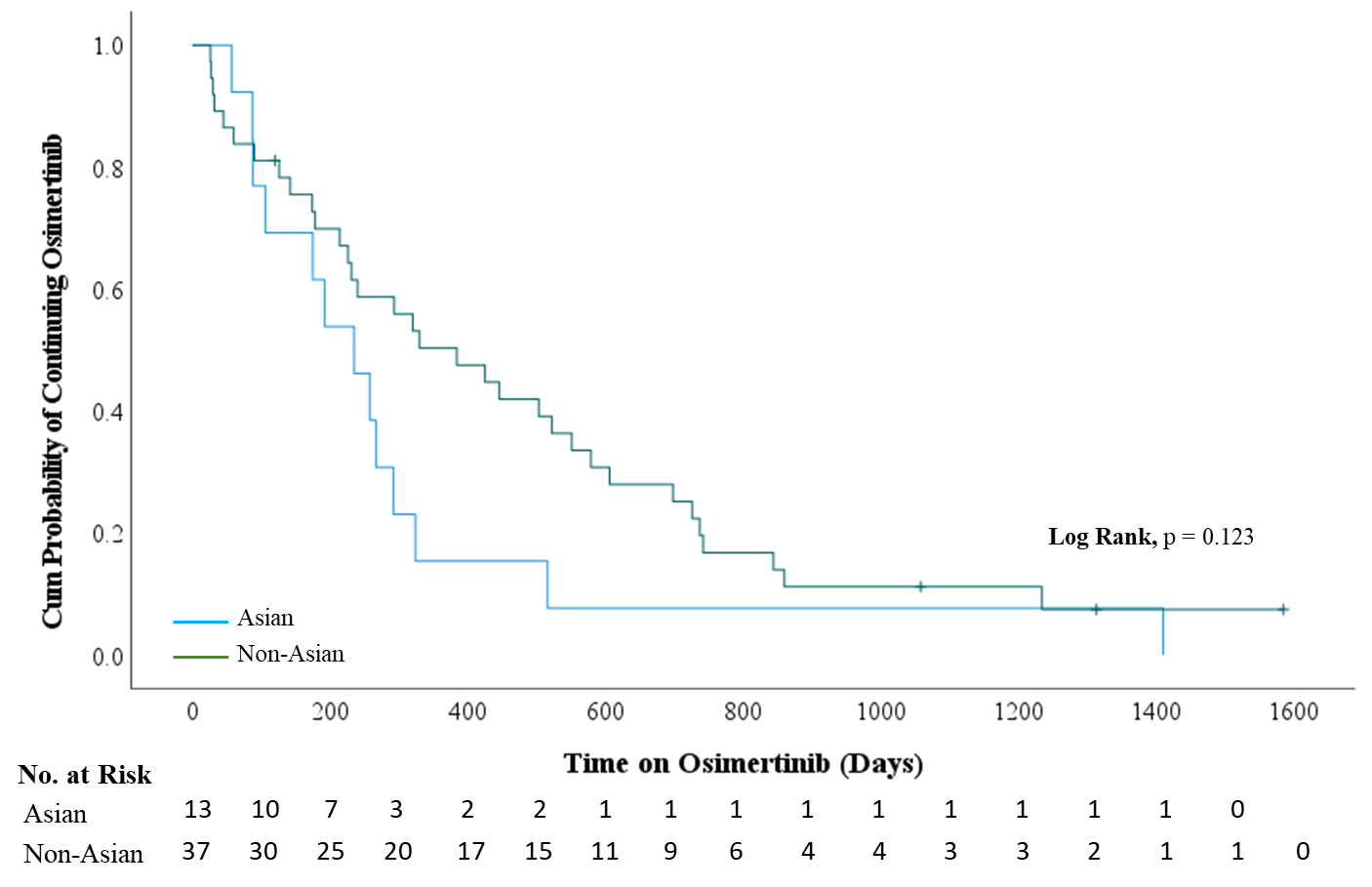

Supplement: Supplemental Materials [file mmc1.docx]
